# Structural evolution and phase transition mechanism of $\hbox {MoSe}_2$ under high pressure

**DOI:** 10.1038/s41598-021-01527-5

**Published:** 2021-11-11

**Authors:** Yifeng Xiao, Shi He, Mo Li, Weiguo Sun, Zhichao Wu, Wei Dai, Cheng Lu

**Affiliations:** 1 Faculty of Materials Science and Chemistry, China University of Geosciences, Wuhan, 430074 China; 2 Department of physics, Stevens Institute of Technology, Castle Point Terrace, Hoboken, 07030 USA; 3 College of Physics and Electronic Information, Luoyang Normal University, Luoyang, 471934 China; 4 School of Mechanical Engineering and Electronic Information, China University of Geosciences, Wuhan, 430074 China; 5 School of Mathematics and Physics, Jingchu University of Technology, Jinmen, 448000 China; 6 School of Mathematics and Physics, China University of Geosciences, Wuhan, 430074 China

**Keywords:** Condensed-matter physics, Structural materials, Theory and computation

## Abstract

$\hbox {MoSe}_2$ is a layered transition-metal dichalcogenide (TMD) with outstanding electronic and optical properties, which is widely used in field-effect transistor (FET). Here the structural evolution and phase transition of $\hbox {MoSe}_2$ under high pressure are systematically studied by CALYPSO structural search method and first-principles calculations. The structural evolutions of $\hbox {MoSe}_2$ show that the ground state structure under ambient pressure is the experimentally observed *P*6$_3$*/mmc* phase, which transfers to *R*3*m* phase at 1.9 GPa. The trigonal *R*3*m* phase of $\hbox {MoSe}_2$ is stable up to 72.1 GPa, then, it transforms into a new *P*6$_3$*/mmc* phase with different atomic coordinates of Se atoms. This phase is extremely robust under ultrahigh pressure and finally changes to another trigonal *R*-3*m* phase under 491.1 GPa. The elastic constants and phonon dispersion curves indicate that the ambient pressure phase and three new high-pressure phases are all stable. The electronic band structure and projected density of states analyses reveal a pressure induced semiconducting to metallic transition under 72.1 GPa. These results offer a detailed structural evolution and phase diagram of $\hbox {MoSe}_2$ under high pressure, which may also provide insights for exploration other TMDs under ultrahigh pressure.

## Introduction

Most transition-metal dichalcogenides (TMDs) are layered compounds, which contain insulators, semiconductors and metals, in which, some of them are superconductors. The molecular formulas of TMDs are $\hbox {MX}_2$, where M is the transition metals, such as W, Mo, Nb, Ta, Ti and others, X is the chalcogen, such as S, Se, Te and so on^1–6^. Up to now, the ground state structures of TMDs under ambient conditions are extensively studied. According to the number of stacked layers, the possible structures of TMDs can be classified into 1T phase with trigonal antiprismatic, 2H phase with trigonal prismatic, 3R phase with trigonal prismatic, etc, which have many stacking patterns in common. Generally, the weak van der Waals force connect layers of TMDs and allow the atom/molecules to enter the interlayers and change their electronic properties^7,8^. On the other hand, pressure can also cause the change of interlayer spacing and the interlayer slip, and lead to the varied structure and electronic properties of different TMDs^9,10^.

$\hbox {MoSe}_2$ is a typical TMD with hexagonal phase stable structure at ambient conditions^1,5^. It is an indirect bandgap semiconductor, with bandgap of about 1 eV. However, there is a very few structural evolutions of $\hbox {MoSe}_2$ under high pressure. In contrast, the structural phase transitions of $\hbox {MoS}_2$ under high pressure are extensively studied. Saha^11^ et al. has carried out the first-principles calculations of $\hbox {MoS}_2$ under high pressure and confirmed the stable high-pressure phases in the pressure range of 100 GPa to 200 GPa, which are *P*4*/mmm* and *I*4*/mmm* structures. Kohulák et al.^12^ has reported that $\hbox {MoS}_2$ transformed from semiconducting to metallic at 40 GPa. However, the interesting subject needs further attentions is that in the similar compound, whether $\hbox {MoSe}_2$ exists the similar pressure induced semiconductor to metal transition.

In the present paper, we focus on the structural transition and electronic properties of $\hbox {MoSe}_2$ under high pressure by using the structure search method and first-principles calculations. Our results show that $\hbox {MoSe}_2$ transfers from *P*6$_3$*/mmc* structure to *R*3*m* phase at 1.9 GPa, which is stable up to 72.1 GPa. Interestingly, as the pressure increase, $\hbox {MoSe}_2$ again transfers from *R*3*m* phase to *P*6$_3$*/mmc*, however, it is metallic, which is different from the semiconducting *P*6$_3$*/mmc* phase under ambient pressure. These results are different from the previous experiments showed that $\hbox {MoSe}_2$ is mostly stable as 2Hc phase below 100 GPa^11,12^. This contradiction leads us to further explore the new phases and structural transition sequence of $\hbox {MoSe}_2$ under high pressure, especially at ultrahigh pressure.

## Theoretical methods

We have conducted a systematical structure search for $\hbox {MoSe}_2$ under high pressure based on Crystal structure AnaLYsis by Particle Swarm Optimization (CALYPSO) approach and first-principles calculations^13–20^. The advantages of these techniques are to predict the stable and metastable structures at the given chemical compositions within certain condition^21,22^. The total energies and electronic properties are calculated within the density functional theory (DFT) framework, as it has implemented by Vienna ab initio simulation package (VASP) code^23^. The projector augmented wave (PAW) method has employed in the DFT calculations to describe electron–ion interactions in $\hbox {MoSe}_2$. The 4*d*$^5$, 5*s*$^1$ and 4*s*$^2$, 4*p*$^4$ are treated as the valence electrons for Mo and Se atoms, respectively^24^. We set the cutoff energy of 600 eV for the wave-function to expand plane waves and select dense Monkhorst–Pack *k*^25^ meshes to ensure all enthalpy calculations are converged in 1 meV/atom. The phonopy code has used to calculate the phonon dispersion curves using 2 $\times$ 2 $\times$ 1 supercells for *P*6$_3$*/mmc*, *R*3*m*, and *R*-3*m* phases of $\hbox {MoSe}_2$^26^. Based on the ground state structures of $\hbox {MoSe}_2$ under different pressure, the energy band structure, density of states, and elastic properties are also calculated^27^ and discussed in detail.

## Results and discussion

**Figure 1:**
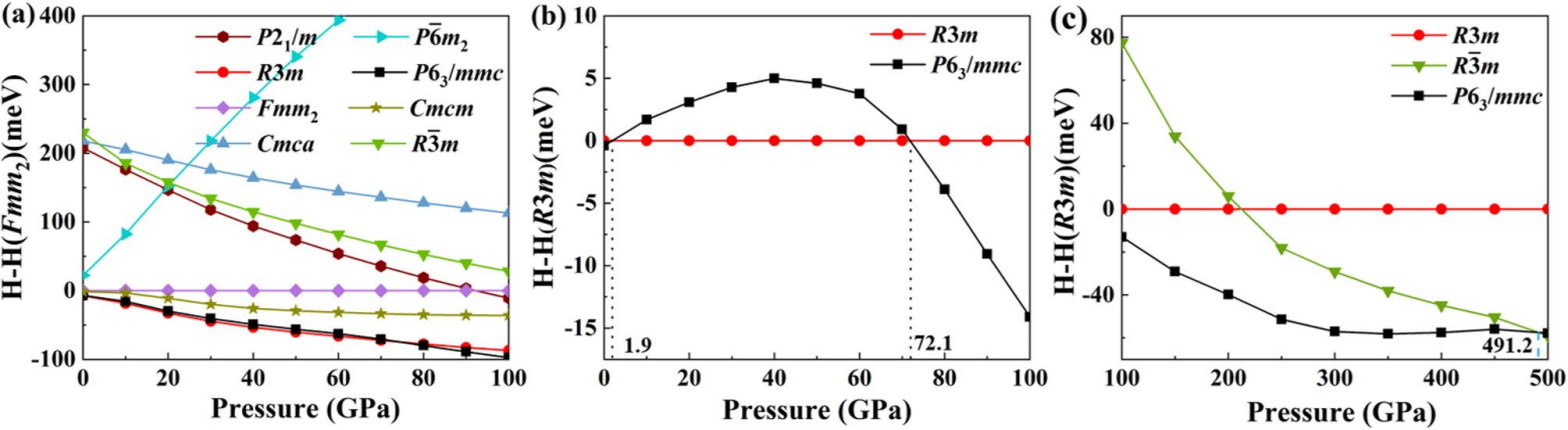
The enthalpy curves of $\hbox {MoSe}_2$ under high pressure. (**a,b**) $\hbox {MoSe}_2$ under high pressure with pressure in range of 0 GPa to 100 GPa. (**c**) $\hbox {MoSe}_2$ under ultrahigh pressure with pressure in range of 100 GPa to 500 GPa.

**Figure 2:**
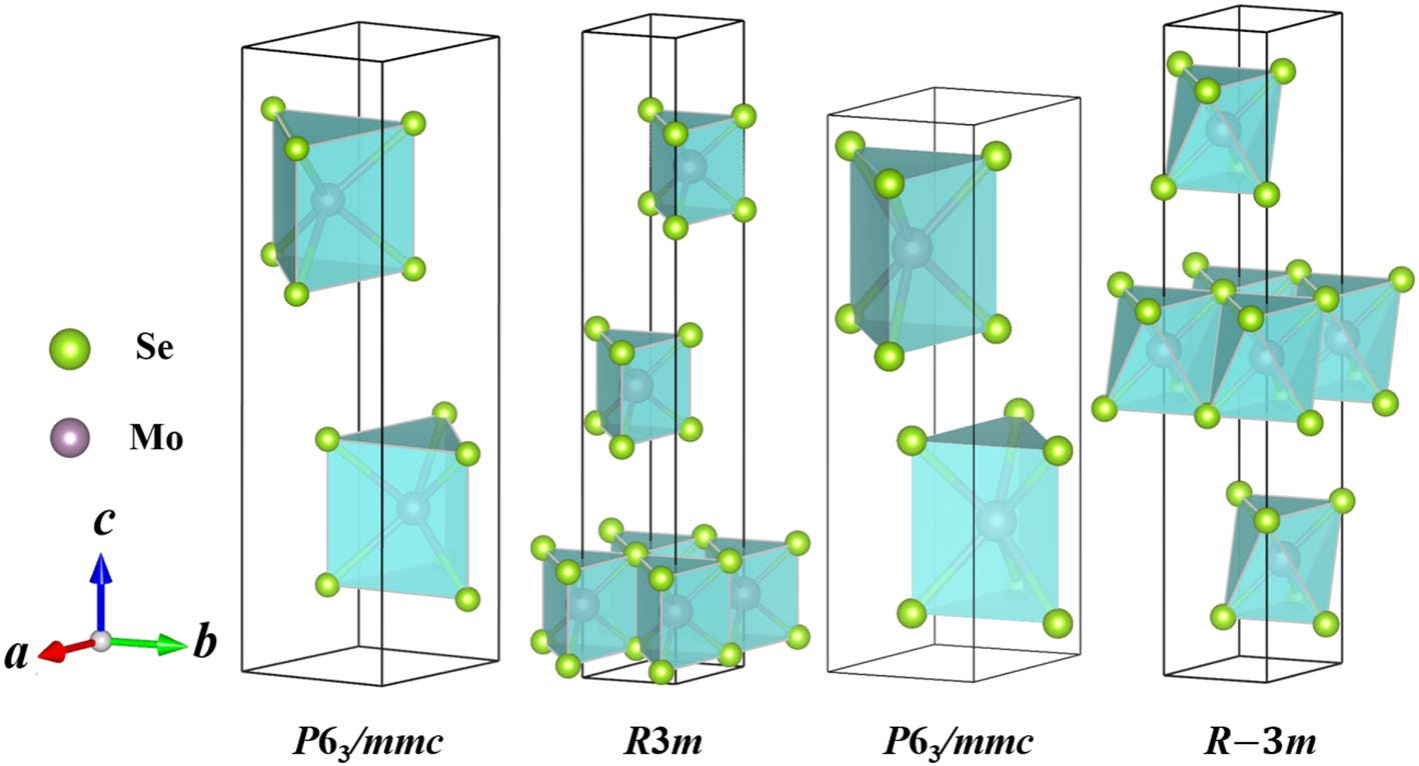
The crystal structures of $\hbox {MoSe}_2$ under high pressure up to 500 GPa. (**a**) *P*6$_3$*/mmc*, (**b**) *R*3*m*, (**c**) *P*6$_3$*/mmc*, and (**d**) *R*-3*m* phases.

We have predicated about 1000 potential structures for $\hbox {MoSe}_2$ at each selected pressure. The top 100 candidate structures of $\hbox {MoSe}_2$ under 0 GPa, 50 GPa, 100 GPa, 200 GPa, and 500 GPa are reoptimized by high accuracy calculations. We have successfully identified the experiment observed *P*6$_3$*/mmc* (2H) phase under ambient pressure, which verifies that the CALYPSO method is perfectly suitable for $\hbox {MoSe}_2$ and the searched results are reliable. It can be seen from Fig. 1a that the enthalpies of *R*3*m* and *P*6$_3$*/mmc* phases are almost the same when the pressure increase from 0 to 100 GPa. Interestingly, some potential low energy phases at low-pressure range are all layered structures. Thus, we have considered the van der Waals (VDW) interactions in the DFT calculations under low-pressure between 0 to 10 GPa. From Fig. 1b, we can clearly find that the energy of *P*6$_3$*/mmc* phase is lower than that of *R*3*m* phase at 0 GPa to 1.9 GPa^28^, and the energy of *R*3*m* phase is lower than that of *P*6$_3$*/mmc* phase with pressure ranged of 1.9 GPa to 72.1 GPa. In fact, the transform pressure of $\hbox {MoSe}_2$ from *R*3*m* phase to *P*6$_3$*/mmc* phase is almost unchanged with/without considering the VDW effects. The transform pressure of $\hbox {MoSe}_2$ from *R*3*m* phase to *P*6$_3$*/mmc* phase is about 2.5 GPa by without considering the VDW interactions, which maybe due to that the Mo and Se atoms are relatively heavy and the influences of VDW interactions on the energy calculations of $\hbox {MoSe}_2$ are negligible. When the pressure is higher than 72.1 GPa, a new *P*6$_3$*/mmc* phase is uncovered, which is different from the initial *P*6$_3$*/mmc* phase. The main differences are the crystal lattice parameters and atomic coordinates of Se atoms. It is extremely robust under ultrahigh pressure and final changes to the trigonal *R*-3*m* phase under 491.1 GPa. The structural phase transition of $\hbox {MoSe}_2$ under ultrahigh pressure is shown in Fig. 1c. The corresponding crystal structures of $\hbox {MoSe}_2$ under high pressure up to 500 GPa are shown in Fig. 2. To further prove the structural stability of $\hbox {MoSe}_2$, we have calculated the formation energies of possible phases and considered the potential energy decomposition to bulk Se and Mo crystals and relevant Mo–Se compounds. The calculations once again indicate that $\hbox {MoSe}_2$ is stable. The detailed results are shown in Fig. S1 in the Supplementary Information.

From Fig. 2, we can find that the unit cell of *P*6$_3$*/mmc* is stacked repeatedly with a period of two $\hbox {MoSe}_6$ layers, while cells of *R*3*m* and *R*-3*m* are stacked repeatedly with a period of three $\hbox {MoSe}_6$ layers. The optimized lattice parameters and atomic coordinates of the four phases are listed in Table 1.

**Table 1:** Calculated lattice constants and atomic coordinates of $\hbox {MoSe}_2$ under selected pressures.

Pressure (GPa)	Structure	Parameter (Å,$\circ$)	Atom	*x*	*y*	*z*
0	*P*6$_3$*/mmc*	*a* = *b* = 3.3226, *c* = 14.3363	Mo1	0.3333	0.6667	0.2500
		$\alpha$ = $\beta$ = 90$^\circ$, $\gamma$ = 120$^\circ$	Se1	0.6667	0.3333	0.6338
20	*R*3*m*	*a* = *b* = 3.1676, *c* = 17.4749	Mo1	− 0.0000	− 0.0000	0.1128
		$\alpha$ = $\beta$ = 90$^\circ$, $\gamma$ = 120$^\circ$	Se1	0.6667	0.3333	0.0166
			Se2	0.6667	0.3333	0.2085
80	*P*6$_3$*/mmc*	*a* = *b* = 2.8898, *c* = 11.0080	Mo1	0.3333	0.6667	0.2500
		$\alpha$ = $\beta$ = 90$^\circ$, $\gamma$ = 120$^\circ$	Se1	0.3333	0.6667	0.5949
500	*R*-3*m*	*a* = *b* = 2.3687, *c* = 15.3738	Mo1	0.3333	0.6667	0.1667
		$\alpha$ = $\beta$ = 90$^\circ$, $\gamma$ = 120$^\circ$	Se1	− 0.0000	0.0000	0.2770

**Figure 3:**
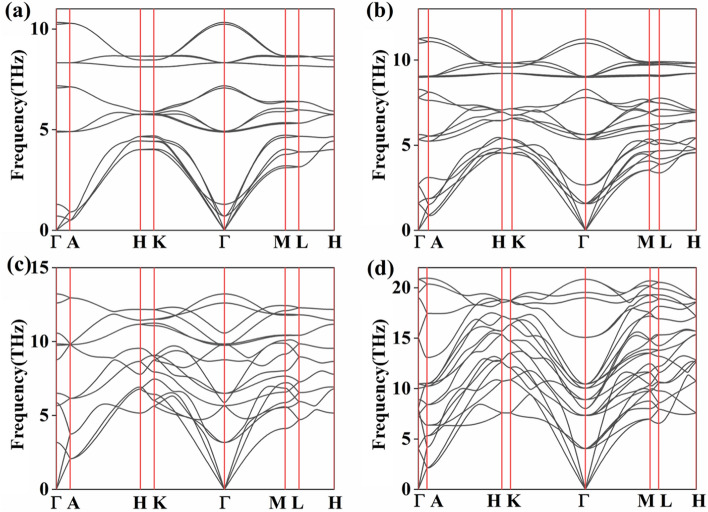
The phonon dispersion curves of $\hbox {MoSe}_2$. (**a**) *P*6$_3$*/mmc* under 0 GPa, (**b**) *R*3*m* under 20 GPa, (**c**) *P*6$_3$*/mmc* under 80 GPa, and (**d**) *R*-3*m* under 500 GPa, respectively.

We now test the chemical, dynamical, and mechanical stabilities of $\hbox {MoSe}_2$. The cohesive energy of $\hbox {MoSe}_2$ can be calculated by the formula as following^29–33^,

1 $$\begin{aligned} E_{coh}=\frac{xE_{Mo}+yE_{Se}-E_{{Mo_x}{Se_y}}}{x+y}, \end{aligned}$$

where $\hbox {E}_{{Mo}}$, $\hbox {E}_{{Se}}$, and $\hbox {E}_{{Mo_x}{Se_y}}$ are the energies of Mo atom, Se atom, and a unit cell of $\hbox {MoSe}_2$, respectively^27,34^. The cohesive energies of the four candidate of $\hbox {MoSe}_2$ ( 0 GPa *P*6$_3$*/mmc*, 20 GPa *R*3*m*, 80 GPa *P*6$_3$*/mmc* and 500 GPa *R*-3*m* ) are − 13.49, − 13.29, − 1.83 and − 5.29 eV per atom, respectively. These results indicate that the bulk $\hbox {MoSe}_2$ is strongly bonded with good chemical stability. Subsequently, we have calculated the phonon dispersion curves of four structures of $\hbox {MoSe}_2$ within different pressures. The results are displayed in Fig. 3. There is no presence of imaginary frequency in the Brillouin zone, which indicates that these four phases of $\hbox {MoSe}_2$ are dynamically stable.

**Table 2:** The calculated the elastic constants of $\hbox {MoSe}_{2}$.

Pressure (GPa)	Structure	$\hbox {C}{_{1}{1}}$ (GPa)	$\hbox {C}{_{1}{2}}$ (GPa)	$\hbox {C}{_{1}{3}}$ (GPa)	$\hbox {C}{_{1}{4}}$ (GPa)
0	*P*6$_3$*/mmc*	173	57	118	58
20	*R*3*m*	267	70	194	100
80	*P*6$_3$*/mmc*	501	145	678	180
500	*R*-3*m*	1659	826	2171	417

Meanwhile, we have calculated the elastic constants of the four phases of $\hbox {MoSe}_2$ under different pressures, which are *P*6$_3$*/mmc* phase at 0 GPa, *R*3*m* phase at 20 GPa, *P*6$_3$*/mmc* phase at 80 GPa, and *R*-3*m* phase at 500 GPa. The elastic constants are listed in Table 2. The stability criteria of hexagonal and trigonal crystal structure are $C_{11} > \vert C_{12}\vert$ , $(C_{11}+C_{12}) > 2C^2_{13}$, $(C_{11}-C_{12})C_{44} > 2C^2_{14}$ for trigonal crystal and $C_{11} > 0$ , $C_{44} > 0$ , $C_{11} > \vert C_{12}\vert$ , $(C_{11}+C_{12}) > 2C^2_{13}$ for hexagonal crystal^35^. According to the above criteria, we note that the calculated elastic constants match well with the stability criteria in corresponding space group symmetries^36–39^. Thus, we can conclude that these four phases of $\hbox {MoSe}_2$ are mechanical stability.

**Figure 4:**
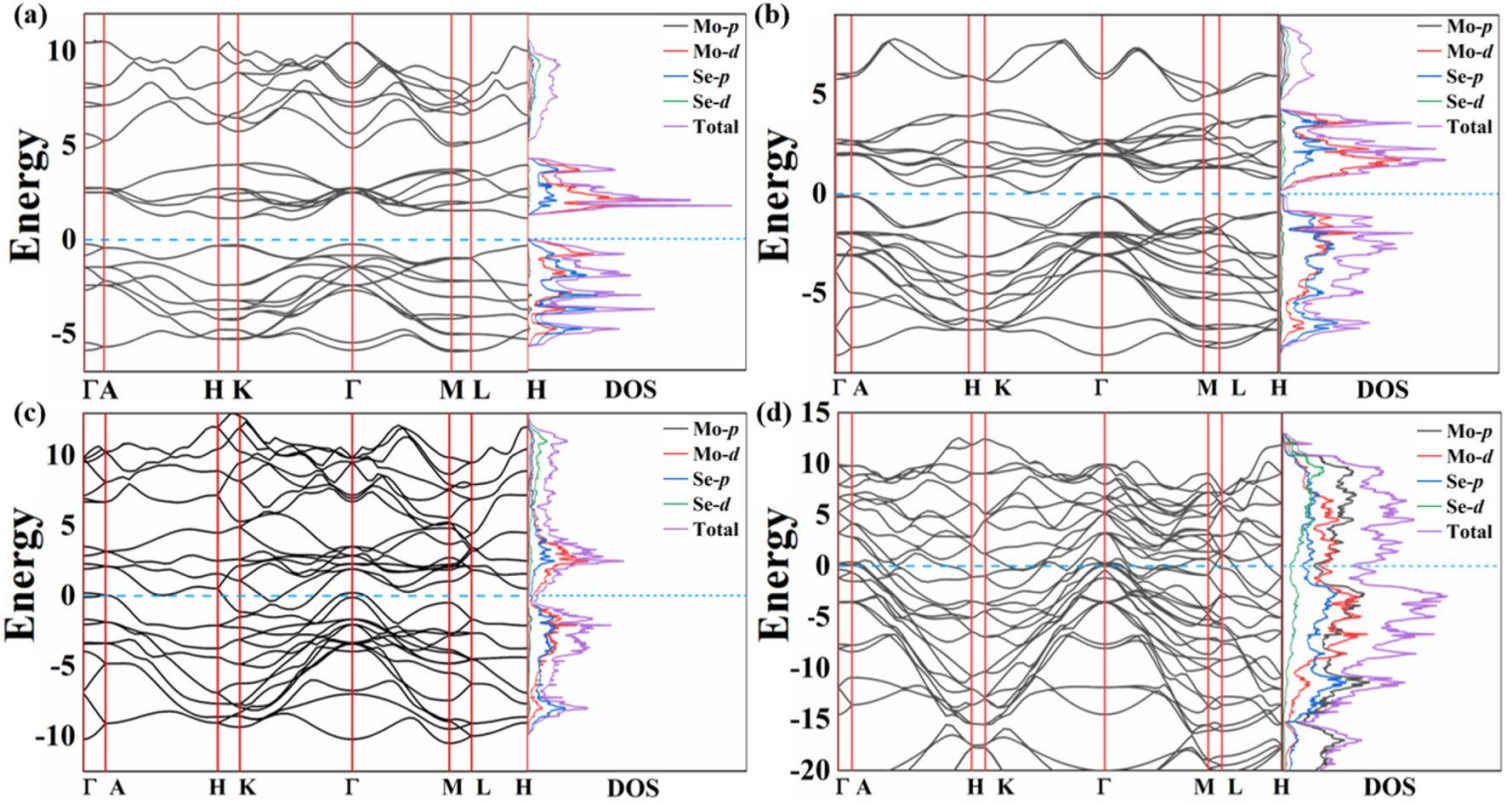
Band structure and projected density of states of $\hbox {MoSe}_2$. (**a**) *P*6$_3$*/mmc* phase at 0 GPa, (**b**) *R*3*m* phase at 20 GPa, (**c**) *P*6$_3$*/mmc* phase at 80 GPa, and (**d**) *R*-3*m* phase at 500 GPa, respectively.

To deeply understand of the effect of pressure on the electronic properties, the evolution of electronic band structure and density of states of the four phases of $\hbox {MoSe}_2$ are shown in Fig. 4. At 0 GPa, the ground state structure is *P*6$_3$*/mmc* phase. It can be seen from Fig. 4a, the *P*6$_3$*/mmc* phase is a direct bandgap semiconductor with bandgap of 1.22 eV. With pressure increasing, the bandgap is slowly decreasing. At 1.9 GPa, the structure *P*6$_3$*/mmc* transforms to *R*3*m* phase^28^, which is an indirect bandgap semiconductor. The bandgap is 0.154 eV under 20 GPa (see Fig. 4b). From 20 to 500 GPa, $\hbox {MoSe}_2$ becomes to a metal as shown in Fig. 4c,d.

The detailed total and partial density of states are calculated (see Supplementary Information, Fig. S3). The states above − 5.5 eV in *P*6$_3$*/mmc* phase at 0 GPa, − 7.5 eV in *R*3*m* phase at 20 GPa, and − 10 eV in *P*6$_3$*/mmc* at 80 GPa are mostly originated from Mo-*d* and Se-*p* orbitals. The Mo-*d* and Se-*p* orbitals show strong *p*-*d* hybridization and indicate obviously covalent bonding characteristics of Mo–Se chemical bond. In *P*6$_3$*/mmc* phase, the orbitals have more overlapping at 80 GPa than 20 GPa, which proves that covalent properties of Mo–Se bond is strengthened by increasing the pressure. In Fig. S3d, we can see a noticeable peak at − 12 eV in the density of states of *R*-3*m* phase at 500 GPa, which are mainly contributed by the *p* orbitals of Mo atoms. Furthermore, except for Mo-*d* and Se-*p* orbitals, the contributions from Mo-*p* orbitals are visibly increased compared with low pressure conditions. This may due to the firmer $\hbox {MoSe}_6$ octahedra in *R*3*m* phase of $\hbox {MoSe}_2$.

**Figure 5:**
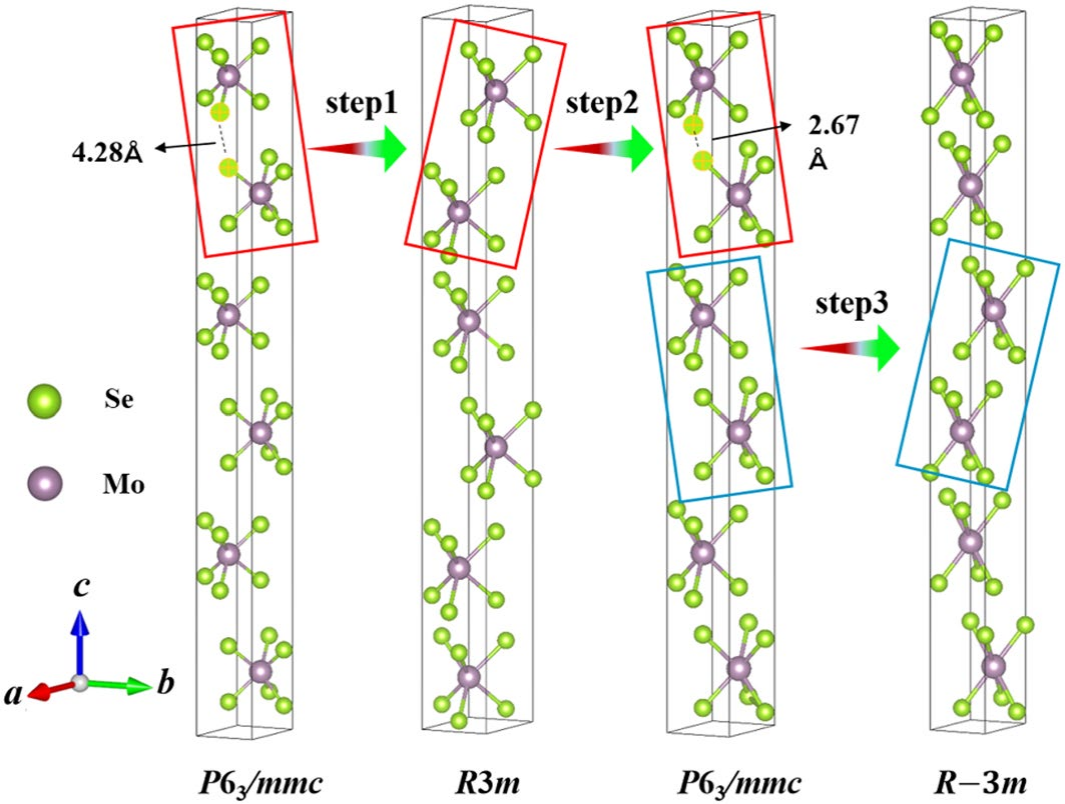
The schematic diagram of four phases of $\hbox {MoSe}_2$ under different pressures in the pressure range of 0 GPa to 500 GPa. (**a**) 1 $\times$ 1 $\times$ 3 supercell for *P*6$_3$*/mmc* phase at 0 GPa, (**b**) 1 $\times$ 1 $\times$ 2 supercell for *R*3*m* phase at 20 GPa, (**c**) 1 $\times$ 1 $\times$ 3 supercell for *P*6$_3$*/mmc* at 80 GPa, and (**d**) 1 $\times$ 1 $\times$ 2 supercell for *R*-3*m* phase at 500 GPa, respectively.

We return again to search the potential structural phase transition mechanisms of $\hbox {MoSe}_2$ under high pressure. To clearly compare the four phases of $\hbox {MoSe}_2$ under different pressure, we have displayed the crystal structure with the same atomic number of Mo and Se stoms by using the supercell of 1 $\times$ 1 $\times$ 3 for *P*6$_3$*/mmc* phase at 0 GPa, 1 $\times$ 1 $\times$ 2 for *R*3*m* phase at 20 GPa, 1 $\times$ 1 $\times$ 3 for *P*6$_3$*/mmc* phase at 80 GPa, and 1 $\times$ 1 $\times$ 2 for *R*-3*m* phase at 500 GPa, respectively. The schematic diagrams are shown in Fig. 5.

From Fig. 5, we find that the structural phase transitions of $\hbox {MoSe}_2$ under high pressure are attributed to the chiral structure transitions of the top two $\hbox {MoSe}_6$ layers marked in red rectangles and the middle two $\hbox {MoSe}_6$ layers displayed in blue rectangles. The evolution of phase transitions is constituted by three steps. In the first step, three-unit cells of *P*6$_3$*/mmc* phase translate into two *R*3*m* unit cell at 1.9 GPa. The main changes occur at the top two $\hbox {MoSe}_6$ layers in *P*6$_3$*/mmc* and *R*3*m* phases, which is a chiral transform of the two $\hbox {MoSe}_6$ layers with mirror symmetry. In the second step, the two-unit cells of *R*3*m* phase return to three *P*6$_3$*/mmc* unit cells, and the central symmetric transformation occurs again on the top two $\hbox {MoSe}_6$ layers. However, the interlayer spacing of the top two $\hbox {MoSe}_6$ layers decreases from 4.28 to 2.67 Å as pressure increasing from 0 to 80 GPa, as shown in the red square of Fig. 5. In the third step, the structure evolution of $\hbox {MoSe}_2$ under ultrahigh pressure is different from the previous two steps. The structural transformation happens at the middle layers of the $\hbox {MoSe}_6$, as shown in the blue rectangles of Fig. 5. The three-unit cells of *P*6$_3$*/mmc* phase return to two *R*-3*m* unit cells, with a chiral structure transition of the middle two $\hbox {MoSe}_6$ layers. Furthermore, it is easy to find that the pressure induced semiconducting to metallic transition of $\hbox {MoSe}_2$ under high pressure, which is mainly attributed to the different stacking modes of the $\hbox {MoSe}_6$ layers in different phases of $\hbox {MoSe}_2$. These results offer important insights for exploration the evolutions of structures and electronic properties of other TMDs at extreme conditions.

## Conclusion

In summary, we have performed comprehensively structure predictions of $\hbox {MoSe}_2$ under high pressure up to 500 GPa by CALYPSO method and first-principles calculations. Three new high pressure phases of $\hbox {MoSe}_2$ are uncovered, and the phase transition sequence follows the order of *P*6$_3$*/mmc* $\rightarrow$ *R*3*m* $\rightarrow$ P6$_3$/mmc $\rightarrow$ *R*-3*m*. The energy band structure calculations indicate $\hbox {MoSe}_2$ are evolution from direct bandgap semiconductor to indirect bandgap semiconductor, eventually, to a metal with pressure increase. These attractively electronic properties are due to the chiral structure changes of the top two $\hbox {MoSe}_6$ layers in $\hbox {MoSe}_2$. The present findings establish the structural phase diagram of $\hbox {MoSe}_2$ under high pressure and describe the evolutions of structures and electronic properties of $\hbox {MoSe}_2$, which offer important insights for exploration other TMDs at extreme conditions.

## Supplementary Information

Supplementary Figures.
