# Supplementary material for: Structural evolution and phase transition mechanism of MoSe2 under high pressure
Source: Sci Rep. 2021 Nov 11;11:22090. doi: 10.1038/s41598-021-01527-5 (PMC8586237; doi:10.1038/s41598-021-01527-5)
Supplement: Supplementary file 1 — Supplementary Figures. [file 41598_2021_1527_MOESM1_ESM.pdf]

Structural evolution and phase transition mechanism of $MoSe_2$ under high pressure

Yifeng Xiao[1], Shi He[1], Mo Li[2], Weiguo Sun[3], Zhichao Wu [4*], Wei Dai [5*] and Cheng Lu[6*]

[1]Faculty of Materials Science and Chemistry, China University of Geosciences, Wuhan, 430074, China

[2]Department of physics, Stevens Institute of Technology, Castle point terrace, Hoboken, USA.

[3]College of Physics and Electronic Information, Luoyang Normal University, Luoyang, 471022, China.

[4]School of Mechanical Engineering and Electronic Information, China University of Geosciences, Wuhan, 430074, China.

[5]School of Mathematics and Physics, Jingchu University of Technology, Hubei, 448000, China.

[6]School of Mathematics and Physics, China University of Geosciences, Wuhan, 430074, China.
E-mail: wuzhichao@cug.edu.cn, daiweiphysics@163.com, lucheng@calypso.cn,

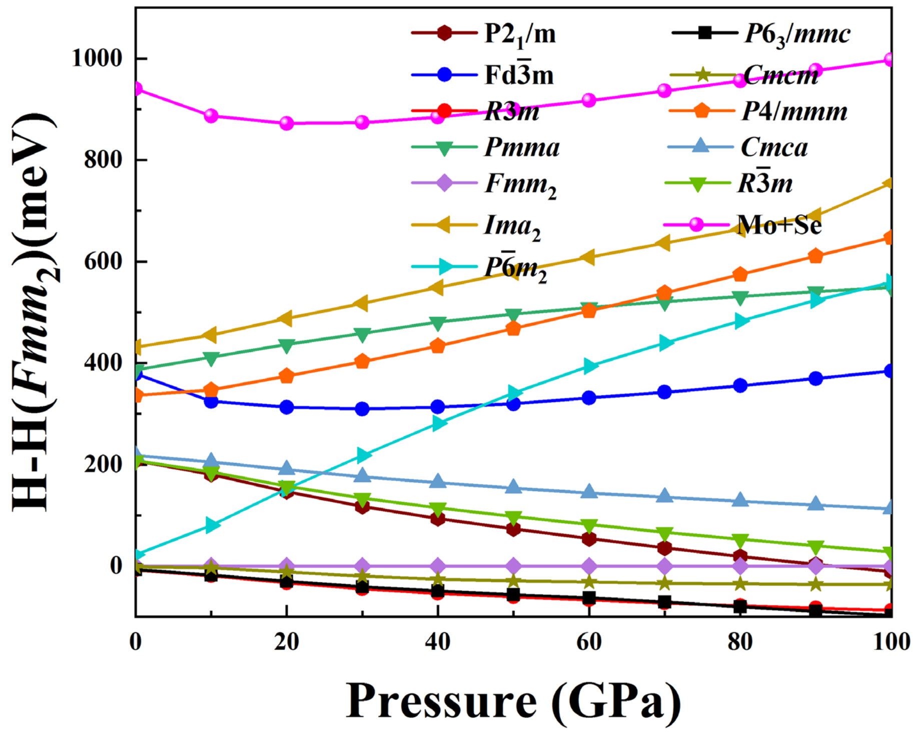

Figure S1: The enthalpy curves of $MoSe_2$ under high pressure. Including decomposition curves of bulk Se and Mo crystals.

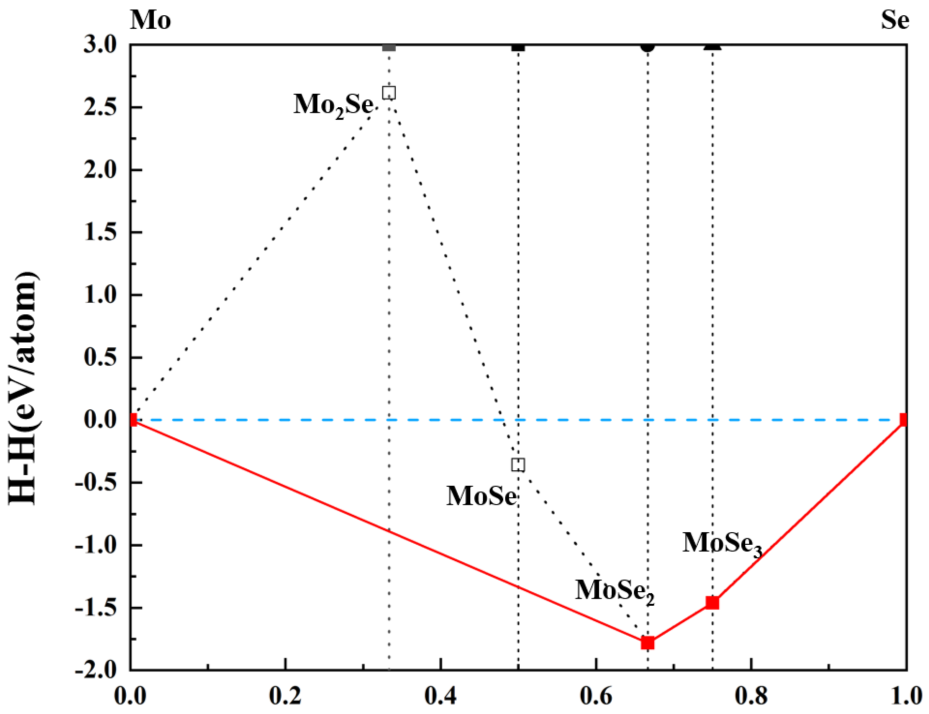

Figure S2: Convex hull of Mo-Se system at 500 GPa.

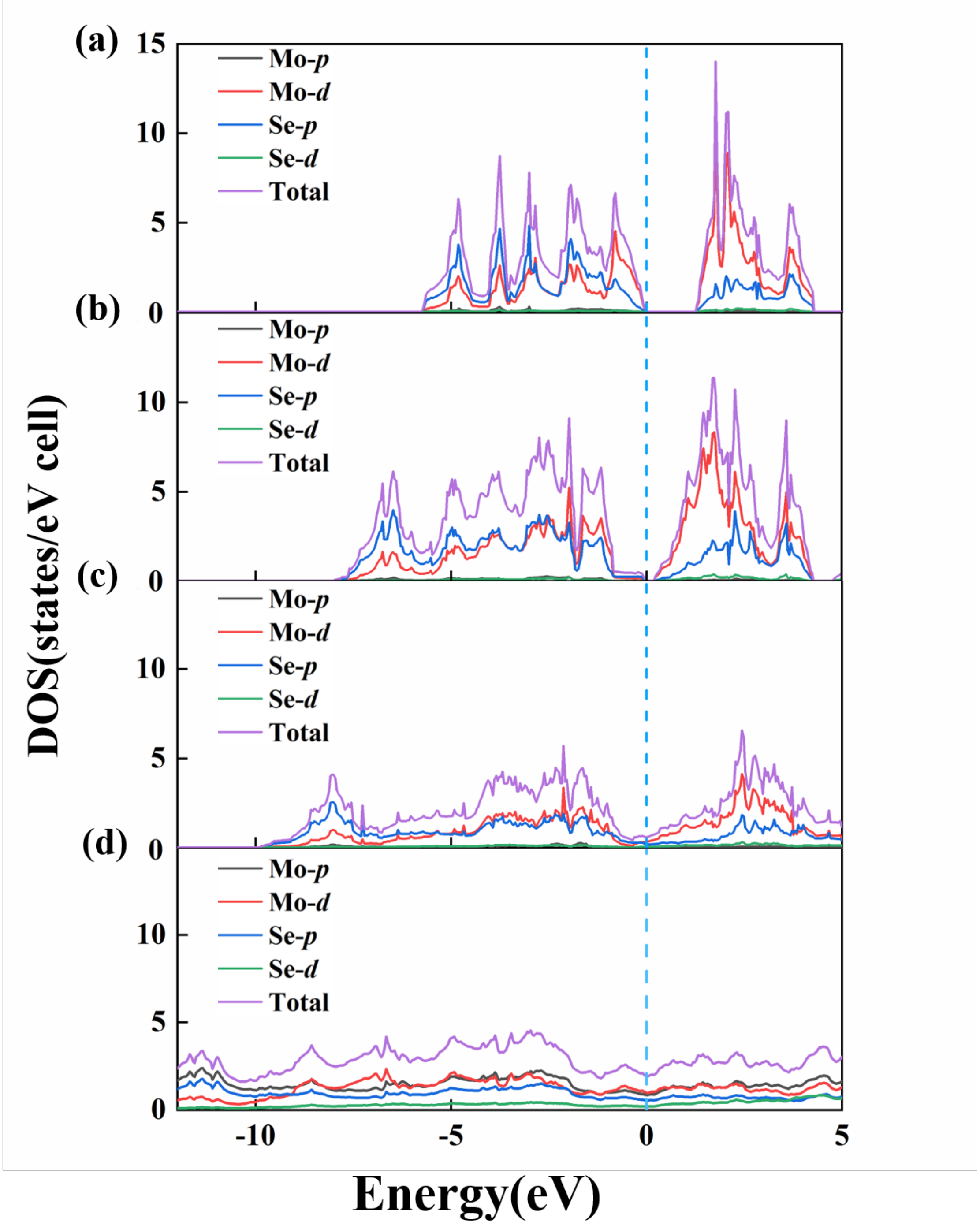

Figure S3: Density of states of $MoSe_2$. (a) *$P6_3/mmc$* phase under 0 GPa, (b) *R3m* phase under 20 GPa, (c) *$P6_3/mmc$* phase under 80 GPa, and (d) *R-3m* phase under 500 GPa, respectively.
